# Clinical Use of the Kannada and English Rate of Reading Tests

**DOI:** 10.3389/fpsyg.2019.02116

**Published:** 2019-09-20

**Authors:** Krithica Srinivasan, James M. Gilchrist, Gopee Krishnan, Arnold Wilkins, Peter M. Allen

**Affiliations:** ^1^Department of Optometry, Manipal College of Health Professions, Manipal Academy of Higher Education, Manipal, India; ^2^Independent Researcher in Optometry and Vision Science, North Yorkshire, United Kingdom; ^3^Department of Speech and Hearing, Manipal College of Health Professions, Manipal Academy of Higher Education, Manipal, India; ^4^Department of Psychology, University of Essex, Colchester, United Kingdom; ^5^Department of Vision and Hearing Sciences, Anglia Ruskin University, Cambridge, United Kingdom; ^6^Vision and Eye Research Unit, Anglia Ruskin University, Cambridge, United Kingdom

**Keywords:** Kannada reading, clinical reference for reading, language development (source: MeSH NLM), language of instruction, reading rate progression, habitual language

## Abstract

The rate of reading test in which randomly ordered common words are read aloud has found use in optometric and educational practice as a means of assessing visual aspects of reading largely independently of comprehension. English and Kannada versions of the rate of reading test were administered to 761 children aged between 9 and 15 years. These children were recruited from four private and five state schools in Udupi Taluk that had English and Kannada, respectively, as the principal medium of instruction. The results showed that the reading rate increased with age, as expected, but depended on the language of the test and the medium of instruction. The study emphasizes the importance of using normative data based on age and the language of instruction used in school. It also suggests age-specific criteria to measure the efficacy of any visual intervention aimed at improving reading speed. The test can be used to assess (1) educational under-performance in reading and (2) the effects of optometric and educational intervention, and separate norms appropriate for each use are presented.

## Introduction

The Wilkins rate of reading test (WRRT) provides an estimate of the rate at which randomly ordered common words can be read aloud. The test is designed to measure visual aspects of reading independently of comprehension. It has proved useful in optometric practice for assessing the effects of interventions such as correction of refractive error (O’Leary et al., 2014), prisms to improve reading speed (O’Leary and Evans, 2006), and colored overlays to reduce visual stress (Wilkins et al., 1996). Versions of the test have been created in various languages, including Kannada (Srinivasan et al., 2018). The Kannada version has been shown to have good reliability and validity (Srinivasan et al., 2018), similar to the English version (Wilkins et al., 1996). The Kannada version shows the expected effects of age on reading rate. However, in order to be useful for clinicians a set of recommended reference or criterion values are necessary, and it is the purpose of this paper to provide such values based on statistical analysis of data from a large sample of school children.

Tests of reading rate (and other measures of performance) may be used in one of two ways. One is to assess *differences between individuals*, in order to make decisions on whether specific individuals are achieving typical “normal” levels of performance. In particular, individuals whose performance falls significantly short of typical levels may need further investigation to see whether they have difficulties that require specific intervention or support. The second use of a test is then to measure an individual’s *response to intervention*; that is, whether a significant change in performance over time occurs in that individual following introduction of an intervention.

For assessment of differences between individuals, what matters is the statistical reliability of the test – which indicates its ability to discriminate between individuals – and estimates of average performance of and variation between individuals in the population. The Kannada and English versions of RRT have shown high reliability. Analysis of data obtained from 799 children aged 7 to 16 years (Srinivasan et al., 2018) yielded test-retest correlations of 0.91 and 0.95 for English and Kannada versions of the RRT, respectively. This article extends the analysis of the same dataset to provide estimates of the lower limits of reading rate that might be expected using the English and Kannada versions of the RRT in children of different ages. These values will inform recommendations on criteria that may be used in practice to identify slow readers whose poor performance may justify further investigation.

For assessment of response to intervention, what matters is the precision of the test – that is, its repeatability over time – because a change in reading rate in response to intervention can only be regarded as significant if it exceeds the typical level of test-retest repeatability. We will present statistical analysis of test-retest differences in the data set described previously (Srinivasan et al., 2018) as a basis for setting criteria for change on English and Kannada versions of the RRT.

## Materials and Methods

### Ethics Statement

The investigations done in the study adhered to the principles of the Declaration of Helsinki. The research protocol was approved by the Research Committee of Manipal College of Health Professions and Ethics Committee of Kasturba Hospital, Manipal. The data were collected in schools that belonged to the same district where the institute was located (Udupi district). Parents of all children provided written informed consent and assent was obtained from children after a verbal and a written explanation of the procedures.

### Participants

The participants included children from 9 schools located in Udupi Taluk (in Udupi district of the Karnataka state in India). The medium of instruction was English in four schools and in the remaining five, it was Kannada. The participants belonged to the 2nd to 9th grades from 4 private and 5 state schools that had English and Kannada, respectively, as the principal medium of instruction. Both males and females from each grade who were present on the day of the assessment were considered for the study. Based on the following inclusion and exclusion criteria, a total of 799 (430 male and 369 female) children were recruited in the age range of 7–16 years.

The inclusion criteria were: (1) the ability to correctly read the words present in both the Kannada and English RRTs and (2) visual acuity of better than or equal to 6/6 and N6. We excluded those children with strabismus, ocular abnormalities (observed on external examination), and those with a known ocular anomaly. The testing rooms were naturally illuminated with daylight that ranged from 600 to 900 lux.

### Procedure

#### Rate of Reading

**FIGURE 1:**
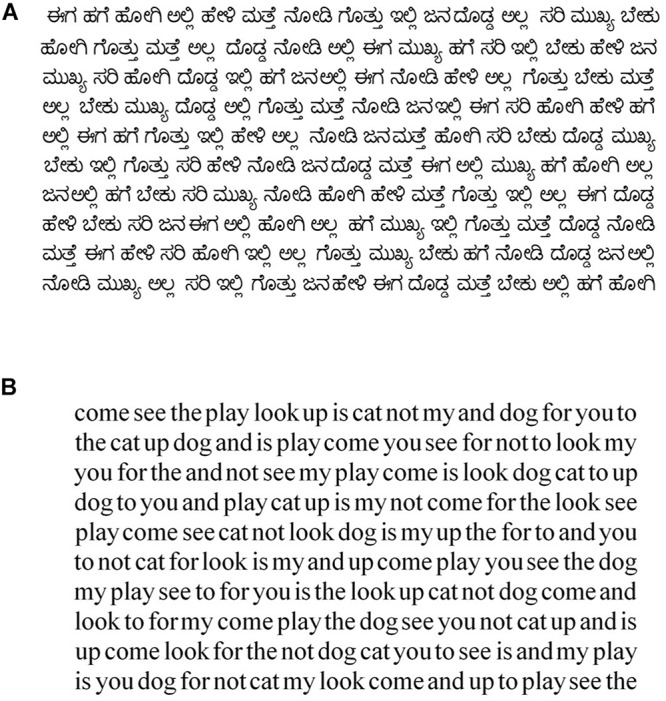
Examples of a Kannada **(A)** and an English **(B)** rate of reading test.

Participants completed the Kannada and English versions of the RRT twice as part of a study that investigated the effects of colored overlay on reading. The results of the overlay will be reported separately in a subsequent paper. The rate of reading was measured without the overlay twice in each language using different versions of the tests that differed only in word sequence (Figure 1). The test RRT was administered to all participants at a viewing distance between 0.4 and 0.5 m.

The order of administration of the English and Kannada RRT versions was alternated across participants with a time interval of approximately 10 min between languages. Each passage was read aloud for 1 min and the number of words correct per minute (wcpm) was recorded. The interval between the two measurements in each language taken on each participant was typically around 5 min. The rate of reading improves with practice (Wilkins and Lewis, 1999), but the practice effects are small, and unaffected by motivational instructions (Wilkins et al., 2001). Any difference between measurements could then be attributed to random variation.

### Statistical Analyses

All analyses were conducted using the free and open statistical platform jamovi^1^.

## Results

### Use of RRT to Identify Atypically Slow Readers

In this section, we examine factors affecting the mean and variability of reading rates among individuals to determine reasonable criteria for identifying slow readers. Reading rate values of individual participants, in the following analysis, were the means of the two measurements taken with each version of the RRT.

A previous article (Srinivasan et al., 2018) presented data obtained using both Kannada and English versions of the RRT, and showed that reading rates of children aged 9–15 years increased with age, as could be expected. However, there were differences between the two versions of the test as well as the medium of instruction (i.e., English vs. Kannada). Prior to analysis, we filtered the original dataset of 761 participants to remove the data that were more than 3 standard deviations from the overall sample mean. This resulted in removal of two individuals, yielding a sample of 759 participants’ data for analysis.

Table 1 presents a statistical analysis of these data – repeated measures (within-subjects) ANOVA for *Test* version, English or Kannada, with medium of *Instruction* as a between-subjects factor, and *Age* as a covariate.

**TABLE 1:** Repeated measures ANOVA: Rate of reading.

	**Sum of squares**	**df**	**Mean square**	***F***	***p***	**Partial η^2^**
**Between-subjects effects**						
Medium of instruction	13549	1	13549	15.3	<0.001	0.020
Age	223382	1	223382	252.2	<0.001	0.250
Residual	669495	756	886			
**Within-subjects effects**						
Test version	172	1	171.9	1.74	0.187	0.002
Test × Medium of instruction	97273	1	97273.2	985.96	<0.001	0.566
Test version × Age	748	1	748.4	7.59	0.006	0.010
Residual	74586	756	98.7			

*Type 3 sums of squares.*

**FIGURE 2:**
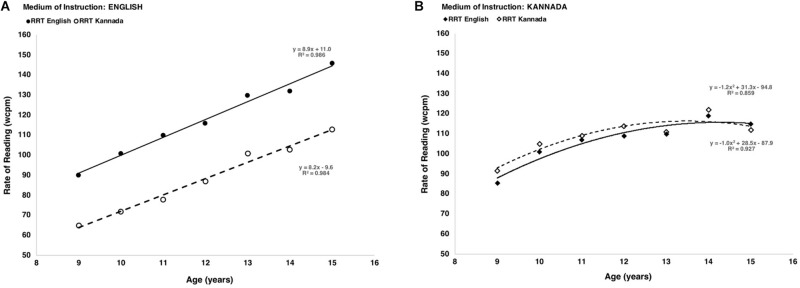
Effect of age and medium of instruction (**A** – English, **B** – Kannada) on rate of reading.

The summary of between-subjects’ effects in Table 1 indicates that both *Age* and *Instruction* have a highly significant influence on rate of reading scores (*p* < 0.001). As shown in the within-subjects summary, differences between versions of the *Test* itself were not significant once the factors of *Age* and *Instruction* were considered (p_Test_ = 0.163), but a significant interaction between the *Test* version and the medium of *Instruction* (p_Test * Instruction_ < 0.001). Therefore, it will be helpful to examine the data further, separating the results according to the medium of instruction. This reveals two distinctly different patterns of performance, as shown in Figures 2A,B.

Figure 2A shows that, for children who are instructed in English, the mean reading rate increases linearly from age 9 to 15 years at a rate of approximately 8 wcpm per year, whether measured by the English version of the RRT or the Kannada version. However, children of every age read approximately 30 wcpm more slowly on the Kannada RRT. For children instructed in English, therefore, judgments of whether they are typical or slow readers will depend upon which language version of the test is used to measure their reading rate.

On the other hand, for children who are instructed in Kannada, Figure 2B shows that there is very little difference in reading rate between English and Kannada versions of the RRT, at any age. Furthermore, the plots for children instructed in Kannada show a ceiling effect, in that reading rates do not increase linearly with age but reach a maximum around age 12, and remain more or less constant thereafter.

**FIGURE 3:**
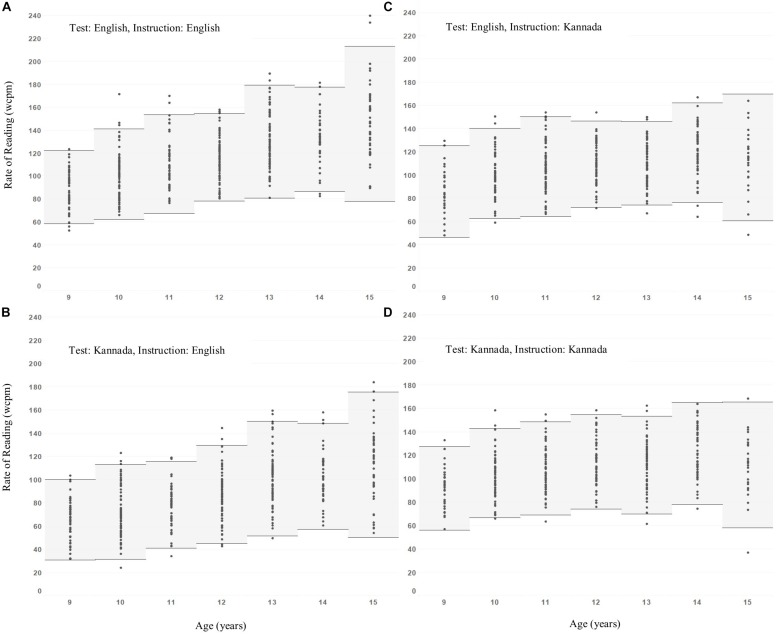
Mean rate of reading measures ±2 standard deviations as a function of age for the groups categorized based on reading test and medium of instruction. **(A)** English test-English instruction, **(B)** Kannada test-English instruction, **(C)** English test-Kannada instruction, **(D)** Kannada test-Kannada instruction.

The differences seen in Figure 2 suggest that if the RRT is to be used to assess differences between children, and identify those who are atypically slow readers, then criteria may need to be different according to which language version of the test is used and in which language children have been taught. To further inform this possibility, therefore, we have examined the distributions of reading rates in the four categories presented in Figure 1, that is *Test*: English and Kannada, and *Instruction*: English and Kannada. Representations of these distributions are in Figures 3A–D.

**FIGURE 4:**
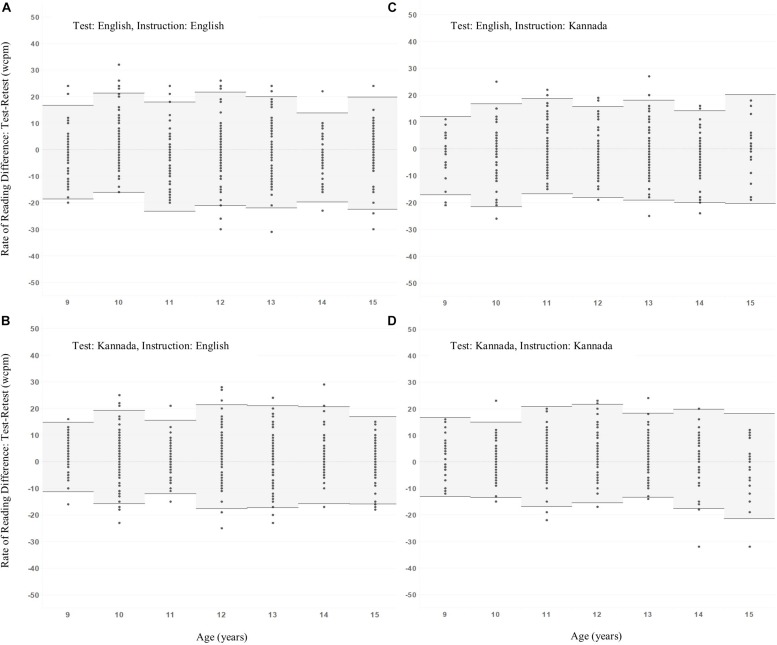
Mean test-retest rate of reading differences ±2 standard deviations as a function of age for the groups categorized based on reading test and medium of instruction. **(A)** English test-English instruction, **(B)** Kannada test-English instruction, **(C)** English test-Kannada instruction, **(D)** Kannada test-Kannada instruction.

Data points in Figures 4A–D show test-retest means for the individuals in each age group, with horizontal bold lines indicating group means, with shaded areas extending ±2 standard deviations either side of the mean. Detailed summary statistics are given in Table 2, including Shapiro-Wilk statistics for normality. Values in Table 2 include the lower 95% limits of variation, which may be used to guide the choice of pass/fail criteria to identify atypically slow readers.

**TABLE 2:** Reading rate summary statistics, by test, medium of instruction, and age.

		**Age**		**N**	**Shapiro-Wilk p**	**Mean**	**SD**	**Mean-2SD**
**Test:**	**English**		**Instruction**	**English**
		9		52	0.594	90.2	15.9	58.4
		10		80	0.025	101.0	19.8	61.4
		11		54	0.007	110.0	21.5	67.0
		12		72	0.276	116.0	19.1	77.8
		13		72	130.0	24.7	19.8	80.6
		14		41	0.749	132.0	22.8	86.4
		15		46	0.092	146.0	33.9	78.2
**Test:**	**English**		**Instruction**	**Kannada**
		9		30	0.866	85.6	19.7	46.2
		10		58	0.878	101.0	19.5	62.0
		11		57	0.417	107.0	21.5	64.0
		12		51	0.796	109.0	18.7	71.6
		13		66	0.904	110.0	18.0	74.0
		14		53	0.810	119.0	21.5	76.0
		15		27	0.639	115.0	27.3	60.4
**Test:**	**Kannada**		**Instruction**	**English**
		9		52	0.710	65.1	17.3	30.5
		10		80	0.272	72.0	20.5	31.0
		11		54	0.526	78.0	18.7	40.6
		12		72	0.778	87.1	21.1	44.9
		13		72	0.443	101.0	24.7	51.6
		14		41	0.440	103.0	22.8	57.4
		15		46	0.555	113.0	31.4	50.2
**Test:**	**Kannada**		**Instruction**	**Kannada**
		9		30	0.909	91.7	17.8	56.1
		10		58	0.622	105.0	19.1	66.8
		11		57	0.821	109.0	20.0	69.0
		12		51	0.332	114.0	20.2	73.6
		13		66	0.585	111.0	20.9	69.2
		14		53	0.293	122.0	21.8	78.4
		15		27	0.704	112.0	26.8	58.4

We note that the standard deviations are broadly consistent across ages and conditions, and so the lower 95% range limits follow a pattern similar to that of the sample means as illustrated in Figure 2 above. Therefore, we make the following suggestions for using the RRT to assess differences between children:

1. Children taught in English should be assessed using the English version of the RRT. This is justified by the fact that reading vs. age profiles of these children for English and Kannada RRT versions are almost identical, with the only difference being that children at every age read the Kannada version approximately 30 wcpm more slowly, so there is no advantage in assessing children with more than one test.
2. Children taught in Kannada should be assessed using the Kannada version of the RRT. This is justified by the fact that reading vs. age profiles for English and Kannada RRT versions are almost identical in both form and rates of reading, so there is no advantage in assessing children with more than one test.
3. If a single criterion value to identify atypically slow readers is sought (for simplicity), then a pragmatic approach would be to adopt a criterion of 70 wcpm for all children, regardless of age or which test is used. Noting that the penalties for adopting a single criterion for all are that: (a) younger children (age 9–10) may have typical lower-limit reading rates of 60–70 wcpm, so a criterion of 70 wcpm would be too high and would result in some/many being incorrectly identified as slow readers; i.e., false-positives, and conversely, (b) older children (age 13–15), especially those taught and assessed in English, may have typical lower-limit reading rates of around 80 wcpm, so a criterion of 70 wcpm would be too low and would result in some/many slow readers being missed; i.e., false-negatives.
4. A more precise proposal, based on the analysis illustrated above (Figures 2A,B) is to relate the reading rate criterion to age. Taking this approach, regression analysis leads to the following suggested criteria (Table 3) for identifying children with atypically low reading rate.

**TABLE 3:** Suggested criteria for minimum typical reading rates, by test and age.

** English RRT (for children taught in English)**							
Age	9	10	11	12	13	14	15
Minimum reading rate criterion	60	65	70	75	80	85	90
**Kannada RRT (for children taught in Kannada)**							
Age	9	10	11	12	13	14	15
Minimum reading rate criterion	65	65	65	65	70	70	70

### Use of RRT to Assess Individual Reading Rate Change Over Time

Whereas the criteria for assessing differences between children must be based upon the statistics of variation between individuals in the population, those for assessing change in individual children over time must be based upon the repeatability of measurements. If an individual provides two measurements on the RRT with just a short time interval between them then as long as there has been no change in the true reading rate of the individual during that interval, for example due to practice or fatigue effects, then any difference between the measurements may be assumed to be due to natural, random variation in test results. Consequently, in order to be considered statistically significant, any true change in reading rate over time must exceed the level of random variation. We may use the values of test-retest differences in a sample of individuals to estimate the range of random variation in the population, and thus set criteria for significant change in reading rate.

Having previously recognized the occurrence of four different profiles for test-retest mean reading rate vs. age, according to *Test* version and medium of *Instruction*, we now analyze the test-retest differences in the same manner, to establish whether this distinction is of consequence for the setting of criteria for change.

Inspection of the data showed that some individuals gave unexpectedly large test-retest differences in reading rate. Therefore, prior to analysis, we filtered the original dataset of 761 participants to remove any whose test-retest difference fell more than 3 standard deviations from the overall sample mean difference. This resulted in removal of fifteen individuals, giving a sample size of 746 for analysis.

Table 4 presents a statistical analysis of test-retest differences, using repeated measures (within-subjects) ANOVA for *Test* version, English or Kannada, with medium of *Instruction* as a between-subjects factor, and *Age* as a covariate.

**TABLE 4:** Repeated measures ANOVA: Rate of reading (test-retest differences).

	**Sum of squares**	**df**	**Mean square**	***F***	***p***	**Partial η^2^**
**Within subjects effects**						
Test	17.4	1	17.4	0.225	0.635	<0.001
Test × Instruction	22.6	1	22.6	0.292	0.589	<0.001
Test × Age	11.1	1	11.1	0.143	0.705	<0.001
Residual	57547.1	743	77.5			
**Between subjects effects**						
Instruction	52.3	1	52.3	0.596	0.440	0.001
Age	137.1	1	137.1	1.561	0.212	0.002
Residual	65258	743	87.8			

*Type 3 sums of squares.*

Results in Table 4 show that there is no significant effect on difference scores of any individual factor, nor is there any significant interaction between factors – see also Figure 4.

Data points in Figure 4 show test-retest differences for the individuals in each age group, with horizontal bold lines indicating group means, with shaded areas extending ±2 standard deviations either side of the mean. Detailed summary statistics are given in Table 5, including Shapiro-Wilk statistics for normality. Values in Table 5 include values for 2 × standard deviation of differences, which may be used to guide the choice of pass/fail criteria to identify statistically significant change in reading rate.

**TABLE 5:** Reading rate differences: Summary statistics, by test, medium of instruction, and age.

		**Age**		**N**	**Shapiro-Wilk p**	**Mean**	**SD**	**2SD**
**Test:**	**English**		**Instruction**	**English**
		9		51	0.466	−1.02	8.84	17.7
		10		78	0.008	2.53	9.34	18.7
		11		53	0.153	−2.75	10.30	20.6
		12		73	0.077	0.15	10.70	21.4
		13		70	0.904	−1.04	10.50	21.0
		14		41	0.644	−3.02	8.41	16.8
		15		45	0.046	−1.29	10.60	21.2
**Test:**	**English**		**Instruction**	**Kannada**
		9		29	0.014	−2.59	7.35	14.7
		10		57	0.879	−2.46	9.56	19.1
		11		55	0.473	0.95	8.86	17.7
		12		51	0.383	−1.31	8.48	17.0
		13		66	0.479	−0.61	9.33	18.7
		14		50	0.864	−2.96	8.59	17.2
		15		27	0.198	−0.07	10.10	20.2
**Test:**	**Kannada**		**Instruction**	**English**
		9		51	0.959	1.73	6.52	17.7
		10		78	0.352	1.69	8.78	18.7
		11		53	0.201	1.75	6.92	20.6
		12		73	0.412	1.84	9.75	21.4
		13		70	0.342	1.86	9.53	21.0
		14		41	0.086	2.41	9.10	16.8
		15		45	0.145	0.42	8.18	21.2
**Test:**	**Kannada**		**Instruction**	**Kannada**
		9		29	0.622	1.79	7.47	17.7
		10		57	0.256	0.65	7.12	18.7
		11		55	0.458	1.91	9.41	20.6
		12		51	0.717	3.02	9.32	21.4
		13		66	0.781	2.41	7.90	21.0
		14		50	0.033	1.10	9.37	16.8
		15		27	0.025	−1.67	9.92	21.2

Using the principle that a criterion value for statistically significant change estimated from a sample of individuals should be at least 2 × standard deviation of test-retest differences (2 × SDD), we see from Table 5 that these values are very similar under all conditions and do not vary with age (see also Table 4 ANOVA). Indeed, pooling these values across ages and conditions justifies use of a single change criterion value of 20 wcpm in all circumstances.

For consistency with previous applications of the RRT, in which reading rate differences and criteria for change have been expressed as percentages of initial rates, we note here that the observed variation in reading rate with age would imply that change criteria expressed as percentages should also vary with age. Thus, by scaling the suggested fixed criterion of 20 wcpm by the reading rate means for different ages (Table 2) we propose the change criteria shown in Table 6.

**TABLE 6:** Suggested criteria for change in reading rate, by test and age.

** English RRT (for children taught in English)**							
Age	9	10	11	12	13	14	15
Change criterion (wcpm)	20	20	20	20	20	20	20
Change criterion (%)	22	20	18	17	16	15	14
**Kannada RRT (for children taught in Kannada)**							
Age	9	10	11	12	13	14	15
Change criterion (wcpm)	20	20	20	20	20	20	20
Change criterion (%)	22	19	18	18	18	17	17

## Discussion

We have presented a clinical test for use in southwest India with children who are taught in Kannada and English. We have provided age-related norms that will determine whether a child is reading more slowly than would be expected for their age. We have also provided norms for test-retest variability essential when the test is used to gauge the effects of an intervention aimed at improving reading speed. “Lack of reading fluency in the early grades creates inefficiencies that affect the entire educational system” (Abadzi, 2008). Therefore, it is important to choose the test appropriate for the medium of instruction and use the age-appropriate norms.

Although reading rate increases with age (Wagner et al., 1993), as might be expected, the rate of increase is different for children taught in Kannada compared to those taught in English. When children are taught primarily in Kannada, the reading speed is similar in both English and Kannada, possibly because of the slower reading of bi-syllabic words at a constant speech rhythm masking the expected faster reading in the familiar language (Verhoeven et al., 2004). When the children are taught primarily in English the rate of reading is consistently slower in the Kannada version at all ages. The reasons for this striking difference in reading rate performance between versions of the test and medium of instruction are not obvious. It is possible that children instructed in English (Figure 2A) may find the script in the Kannada RRT to be more visually demanding in terms of attending to and isolating individual words, which would result in a lower rate of reading at every age as more time is needed to process the visual information. It is also possible that there is a significant verbal effect because there are more syllables in the words of the Kannada RRT than in those of the English version. Thus, it is possible that the lower rate of reading on the Kannada RRT may reflect time taken to say the words rather than to see them, but when interpreting these data it is important to bear in mind the socioeconomic differences between children attending schools with Kannada as a primary language of instruction and children taught in English (Dreze and Kingdon, 2001; Baker et al., 2002; Borooah and Sriya, 2005). Children attending the private schools were taught primarily in English and children attending the public schools were taught primarily in Kannada.

The design principles of both the English and the Kannada rate of reading tests are such that rate of reading is not affected by vocabulary skills or language proficiency. Both the tests were designed using words common in each language (high frequency of occurrence) and the words are arranged in a random order in the test. It was designed to assess the visual aspects of reading irrespective of the semantic and syntactic knowledge. Also, one of the inclusion criteria was the ability to read correctly all the words present in both the English and Kannada tests. Therefore, a language proficiency test was not included in this study.

The “rhythm” of a typeface can be assessed using horizontal autocorrelation, the correlation of an image with a second version of itself, displaced horizontally by a small amount (lag). The mathematical technique can be understood by imagining text printed on the transparency of an overhead projector. When two identical transparencies are superimposed and in register the light transmitted through them is at its maximum (the correlation is 1.0). When the top transparency is moved horizontally across the lower one the lag increases and the transmitted light decreases (the correlation decreases), reaching a minimum when a majority of letter strokes on the top transparency are in the spaces between letters on the lower transparency. As the top transparency is moved further, and the lag increases further, the transmitted light increases (and the correlation increases), reaching a maximum when the majority of letter strokes on one transparency lie on the neighboring letter strokes on the other.

The similarity in the spatial periodicity of adjacent strokes results in high auto-correlation. The first peak in an autocorrelation analysis represents the similarity between adjacent strokes within a letter or neighboring letters. The second peak represents the similarity between neighboring letters. The speed with which words are read has been shown to depend upon the similarity between neighboring strokes of letters. Wilkins et al. (2007) showed that the height of the peak predicted the speed with which the word could be read aloud. Words with high peak were read about 10% more slowly than those with low. The peak also predicted the speed of silent visual search through a paragraph of text. Reducing the autocorrelation by compressing words near the middle and expanding them at the edges (leaving their length unchanged) increased reading speed (at least in poor readers), even though the readers preferred to read an undistorted version of the text (Wilkins et al., 2007). One of the reasons for the effects of spatial periodicity on reading speed concerned the ways in which the eyes move across text when reading.

During a rapid eye movement (saccade), one eye generally leads and the other follows, resulting in a misalignment of the eyes that requires correction when the eyes come to rest (Liversedge et al., 2006a, b). Jainta et al. (2010) measured saccades and vergence movements when their participants read German sentences. When the words had a high first peak in the horizontal autocorrelation the reading of sentences was slower because the eyes rested on each word for longer during the vergence eye movements that corrected the misalignment (vergence error). The realignment took longer with a spatially periodic word because the alignment was then more precise.

**FIGURE 5:**
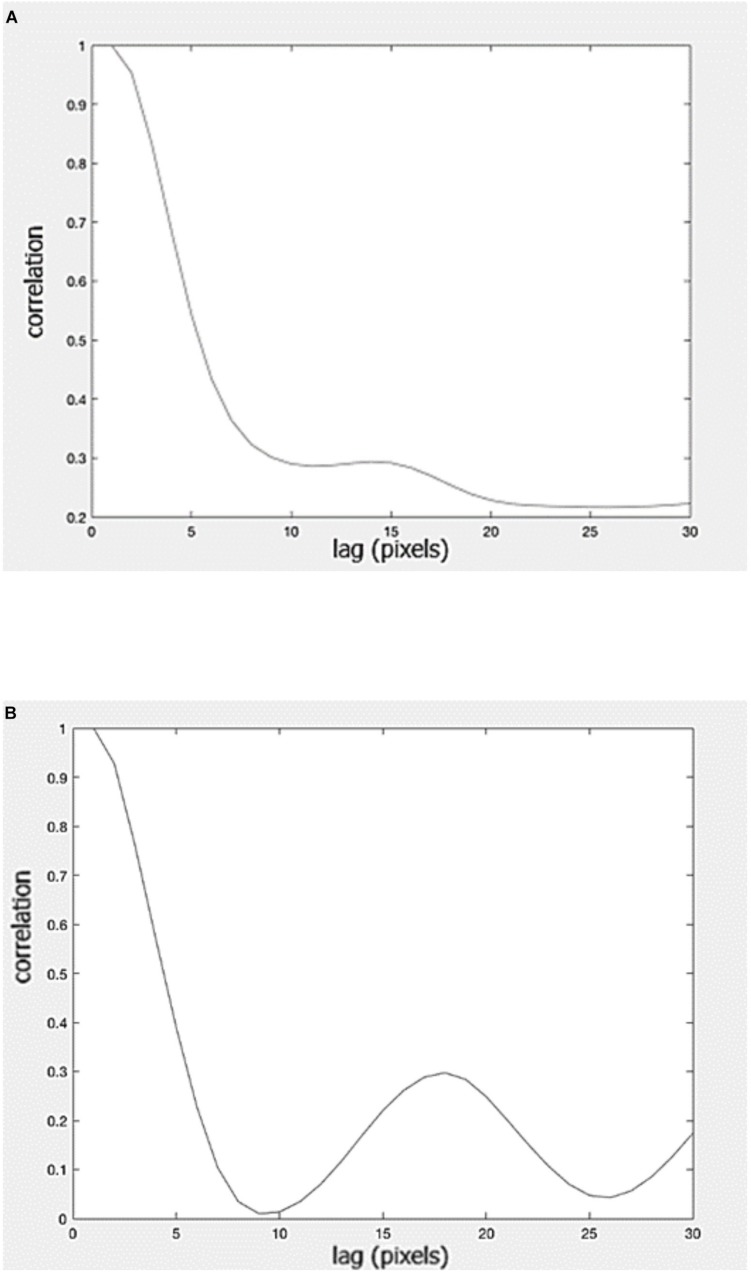
Autocorrelation for the entire **(A)** Kannada and **(B)** English versions of the rate of reading test.

Figure 5 shows that the autocorrelation of the English version (in Times font) is considerably greater than for the Kannada (in Tunga font). Clearly the differences in the spatial rhythmicity of the orthographies and fonts are not sufficient to explain the differences in the absolute reading speed obtained using the English and the Kannada RRTs; they are not matched for factors such as word length, syllable length, and orthographic consistency.

The age-related norms we have presented here will find use in identifying children who are behind in reading speed, with the implication that vision may be a possible contributing factor. If any child has a reading score below the normal values listed herein it is clearly advisable that their vision is thoroughly assessed. The authors would recommend a comprehensive eye examination including objective and subjective refraction and a full binocular vision work-up, with a correction of any significant refractive error, or binocular vision anomaly. The norms for changes in reading speed will provide a measure of the efficacy of any visual intervention aimed at improving reading. It is important to consider the suggested age-wise criteria for defining a change with intervention that is not due to random variation. This study investigated a Kannada RRT based on the original English Wilkins RRT. When the Wilkins RRT is adapted into new languages then a similar study should be conducted in order to produce meaningful clinical norms for that test. One limitation of the study is that the illumination level was not standardized across schools as we aimed to conduct the reading tests under the existing illumination.

## Data Availability

All datasets generated for this study are included in the manuscript and/or the supplementary files.

## Ethics Statement

The investigations done in the study adhered to the principles of the Declaration of Helsinki. The research protocol was approved by the Research Committee of Manipal College of Health Professions and Ethics Committee of Kasturba Hospital, Manipal. Parents of all children provided written informed consent and assent was obtained from children after a verbal and a written explanation of the procedures.

## Author Contributions

KS, GK, and PA conceptualized the research idea and devised the research. KS collected the data and implemented the research idea under the supervision of GK and PA. KS, JG, AW, and PA contributed to the data analysis and interpretation. PA, JG, and AW were involved in the manuscript preparation. All authors contributed to the final review of the manuscript.

## Conflict of Interest Statement

AW receives emoluments based on sales of the Wilkins rate of reading test. The remaining authors declare that the research was conducted in the absence of any commercial or financial relationships that could be construed as a potential conflict of interest.

## Footnotes

1 https://www.jamovi.org/
